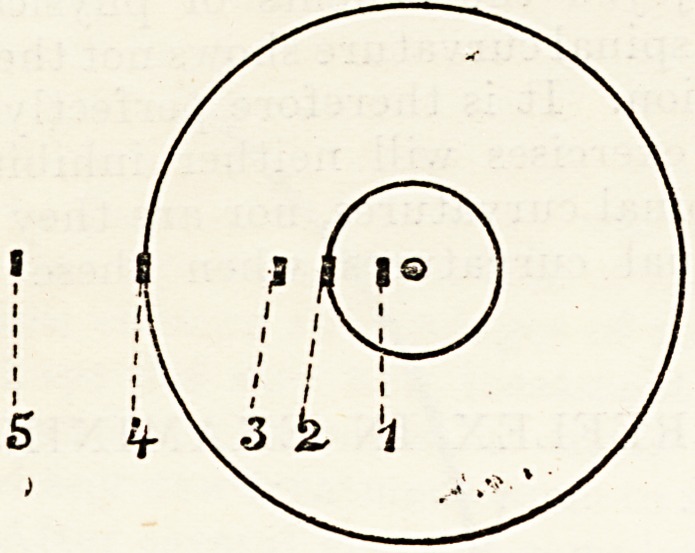# On the Practical Value of the Corneal Reflex in Examining the Eyes of Children

**Published:** 1906-07-07

**Authors:** Sydney Stephenson

**Affiliations:** Ophthalmic Surgeon to the Kensington General Hospital, the Evelina Hospital, the North-Eastern Hospital for Children, and Queen Charlotte's Hospital, London.


### ON THE PRACTICAL VALUE OF THE CORNEAL REFLEX IN EXAMINING THE EYES OF CHILDREN.

By Sydney Stephenson, Ophthalmic Surgeon to the Kensington General Hospital, the Evelina Hospital, the North-Eastern Hospital for Children, and Queen Charlotte's Hospital, London.

In examining the eyes of children, objective methods are of great practical importance, and, indeed, in that class of patient may be said to possess an almost exaggerated value. This somewhat obvious fact has led me to wonder why one of the simplest methods of examining the eyes with the ophthalmoscope—namely, the investigation of the corneal reflex—has received comparatively little attention from practitioners, and appears to be almost unknown outside the ranks of the ophthalmic surgeons. This plan, as will be explained immediately, gives invaluable information, more especially with regard to the deviations and the fixation of the eye.

There is little excuse for neglecting the method (as simple as it is valuable), which was first employed by Priestley Smith,[1] in conjunction with his tape measure, as a means of measuring the deviation in cases of strabismus. A few years later Ernest E. Maddox[2] carried the matter much further, and insisted upon the wide applicability of the corneal reflex test, especially in children. Priestley Smith[3] at about the same time described the employment of the corneal reflexes in ophthalmology, more especially as a test of fixation and deviation of the eyes. As he then justly observed, "the systematic observation of the corneal reflex commonly occupies only a few seconds, and often gives important information which could not be obtained so rapidly in any other way." In a more recent publication[4] the same writer has elaborated still further the applications of the test.

*Method and Applications.*—The investigation of the corneal reflexes with the ophthalmoscope occupies but a few seconds, is simplicity itself, and should, I think, form an integral part in the routine examination of children's eyes.

The surgeon places his hand upon the child's head, for the twofold purpose of steadying the patient and of maintaining a more or less constant distance between himself and the child. Then, holding the ophthalmoscope in his disengaged hand, he throws the light of the larger mirror on to one of the patient's eyes, and notes attentively the position with regard to the pupil occupied by the tiny spot of bright light mirrored upon the cornea. By a quick rotation of the instrument the light is next thrown upon the patient's other eye, and the two observations are contrasted mentally. If the direction of the eyes be normal, the corneal reflex will occupy a corresponding position in the two eyes—that is to say, usually a little to the inner (nasal) side of the centre of the pupil. In that event we may be practically certain that no squint exists, a point now and then difficult to ascertain in any other way. Why does the reflex normally occupy not the centre of the cornea but a position slightly to the nasal side of that point? This is due to the fact that the line of vision—or, rather, the line of fixation—cuts the cornea somewhat to the inner side of its centre.

When a squint is present, the corneal reflex of the deviating eye will be displaced in a direction opposite to that of the strabismus. Thus, in convergence, the reflex lies somewhere to the outer side of the centre of the pupil; in divergence to the inner side of that point; in upward squint, below the centre; and in downward squint, above the centre.

The corneal reflex, moreover, affords a rough idea of the degree of a squint. Clearly, the greater the deviation of the eye, the farther from the centre will the reflex lie. This point is of importance in the out-patient room, where preference must always be given to simple and expeditious methods of clinical examination. The degree of strabismus can be given correctly enough by marking on a rough diagram the exact position occupied by the reflex in the deviating eye, or by translating the same fact into words. It is easy for that matter, once we know the position occupied by the corneal reflex to convert our knowledge into terms of the angular measurement of the squint. Empirically, it has been shown by Hirschberg[5] that a certain deviation corresponds roughly with a certain angular measurement. According to the position of the reflex, Hirschberg distinguishes five grades of strabismus, as follows:—

1. Reflex considerably nearer centre of pupil than edge = $5^\circ$ or $6^\circ$, and certainly less than $10^\circ$.
2. Reflex at edge of pupil (3 mm.) = $12^\circ$ to $15^\circ$.

3. Reflex midway between edge of pupil and edge of cornea = 25°.
4. Reflex on sclero-corneal junction = 45° to 50°.
5. Reflex between cornea and equator of eyeball = 60° to 80°.

An inspection of the accompanying figure (taken from Hirschberg's original communication) will render the foregoing details clear.

Examination of the corneal reflex, however, is capable of rendering still further information. A squinting eye, as well known, may or may not "fix"—*i.e.* when the sound eye is covered the squinting eye may or may not be capable of looking straight at an object held in front of it. Broadly speaking, the power of fixation depends upon the sight possessed by the eye, and that, again, depends upon how long the squint has lasted, and especially upon the age of onset. In order to test this point (one of some importance in view of the surgical treatment to be adopted), the non-squinting eye is covered, and the child told to fix the mirror with the other eye. If fixation be good, the reflex will at once occupy the approximately central portion of the cornea and remain in place, whereas if true fixation be absent, the eye will wander aimlessly about, a point that can be readily appreciated by the vacillating corneal reflex.

The distinction between a comitant squint, on the one hand, and a paralytic squint, on the other, is not always easy to determine in young children, although much may turn upon the diagnosis. In the former the squinting eye, of course, retains its full range of movement, while in the latter the excursions are defective in one or more directions. There is no better way of determining the point than by the corneal reflexes, especially when the paresis is trifling. The *modus operandi* is as follows: "Holding the ophthalmoscope in the usual way with the right hand, lay the palm of the hand on the patient's head, with instructions to let the head follow the most gentle guidance of the hand without any resistence in the neck. Now bid the patient look at the central aperture of the mirror while the light is thrown on the squinting eye, and the exact position of the corneal reflex noted. Now slowly turn the head to the right and left, then up and down, etc., and notice if the position of the reflection is unchanged by these manœuvres; if it is unchanged, the squint is concomitant. It is true that such observations require a good deal of practice before certainty can be acquired, but the same is true of retinoscopy, ophthalmoscopy, etc." (Maddox).

One of the important clinical distinctions between paralytic and comitant squint is that in the former the primary deviation is smaller than the secondary, while in the latter the deviations are equal. This point can in no way be determined with greater readiness than by a comparison of the corneal reflexes.

Gullstrand [6] has advocated the utilisation of the corneal reflexes as a means of diagnosing paresis of the ocular muscles. The method essentially consists in a systematic comparison of the corneal reflexes when the eyes are rotated in the various meridians of the fields of fixation. But it is no part of the present paper to enter into the details of Gullstrand's ingenious though complicated scheme, but rather to point out the simpler clinical applications of the test.

Finally, the reflex is capable of affording information with respect to the exact position of the cornea traversed by the visual axis, or, more correctly, of the line of fixation. This observation may be useful when the placing of an optical iridectomy is in contemplation or when one is engaged in attempting to estimate the effect upon sight of a blemish upon the cornea. The last application is to assure oneself that fixation (and presumably sight) is present in the eyes of a young baby.

After what has been said, it may be fairly concluded that an inspection of the ophthalmoscopic corneal images should form part and parcel of the systematic examination of children's eyes.

[1] Ophthal. Rev., Vol. VII., 1888. [2] Edin. Med. Journ., Jan. 1892. [3] Ophthal. Rev., Vol. XI., 1892, p. 37. [4] IXᵉ Congrès International d'Ophtamologie d'Utrecht, 1899. [5] Centralblatt f. prak. Augenheilkunde, 1886, p. 5. [6] Swedish Academy, Vol. XVIII., Part IV., No. 5, abstracted in Ophthal. Rev., Vol. XI., 1892, p. 359.

## Figures and Tables

**Figure f1:**